# True Exfoliation: Rarity of Degeneration

**DOI:** 10.7759/cureus.100693

**Published:** 2026-01-03

**Authors:** Nga Yan Li, Wing Man Ho, Anita Li, Ching Ki Fung, Lok Man Tiffany Yeung, Kai Wang Kenneth Li

**Affiliations:** 1 Department of Ophthalmology, United Christian Hospital, Hong Kong, HKG; 2 Department of Anatomical and Cellular Pathology, United Christian Hospital, Hong Kong, HKG

**Keywords:** capsulorrhexis, cataract, degeneration, optical coherence tomography, true exfoliation

## Abstract

True exfoliation is the condition in which the anterior capsule layers are delaminated. This case report presents a woman in her 70s with cataract and age-related degenerative true exfoliation. Findings from anterior segment optical coherence tomography (ASOCT) demonstrated a lamellar separation of the anterior portion of the lens capsule typical of true exfoliation. Early recognition of the condition via preoperative and intraoperative ASOCT is vital for better surgical planning and delivery so as to avoid complications in capsulorhexis execution.

## Introduction

True exfoliation is the condition in which the anterior capsule layers are delaminated. First reported by Elschnig in glassblowers [1], various causes, including age-related degeneration, intense heat, ocular trauma, and intraocular inflammation, could be attributed to true exfoliation due to direct damage of the capsular cells or indirect activation of lens capsule proteolysis [2,3]. This case report presents a patient with cataract and age-related degenerative true exfoliation. We evaluate the clinical and pathological features of true exfoliation and discuss the use of anterior segment optical coherence tomography (ASOCT) to facilitate an uneventful phacoemulsification. Findings from ASOCT demonstrate a lamellar separation of the anterior portion of the lens capsule typical of true exfoliation [2]. Unlike pseudoexfoliation syndrome, which is a systemic disease involving the accumulation of fibrillary materials in the anterior segments of the eye, true exfoliation is the result of mechanical stress and degenerative changes to the lens capsular cells, which subsequently lead to anterior lens capsule delamination. Adequate awareness of this condition can facilitate thorough surgical planning and avoid potential capsulorhexis-related complications such as partial-thickness capsulorhexis, radial tear, and radial extension during surgery [4].

## Case presentation

**Figure 1:**
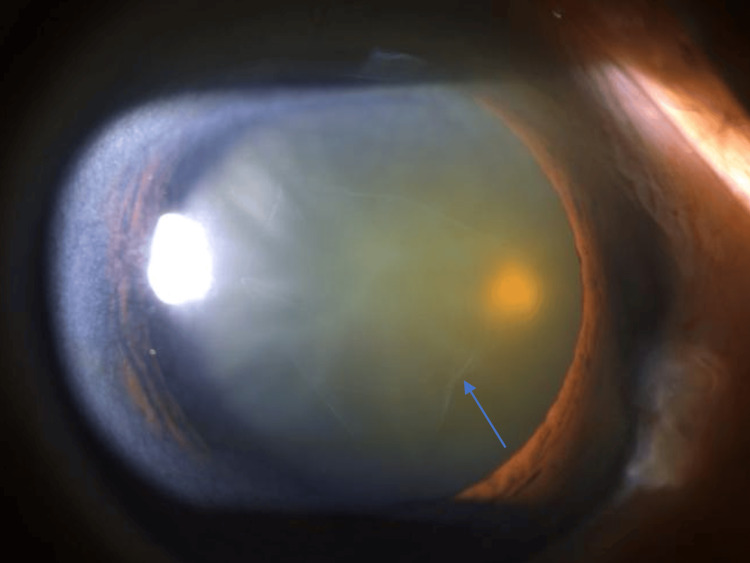
Clinical Photo of the Right Eye Clinical slit lamp photo of the right eye of the patient showing a free-floating membrane in the anterior chamber (arrow).

**Figure 2:**
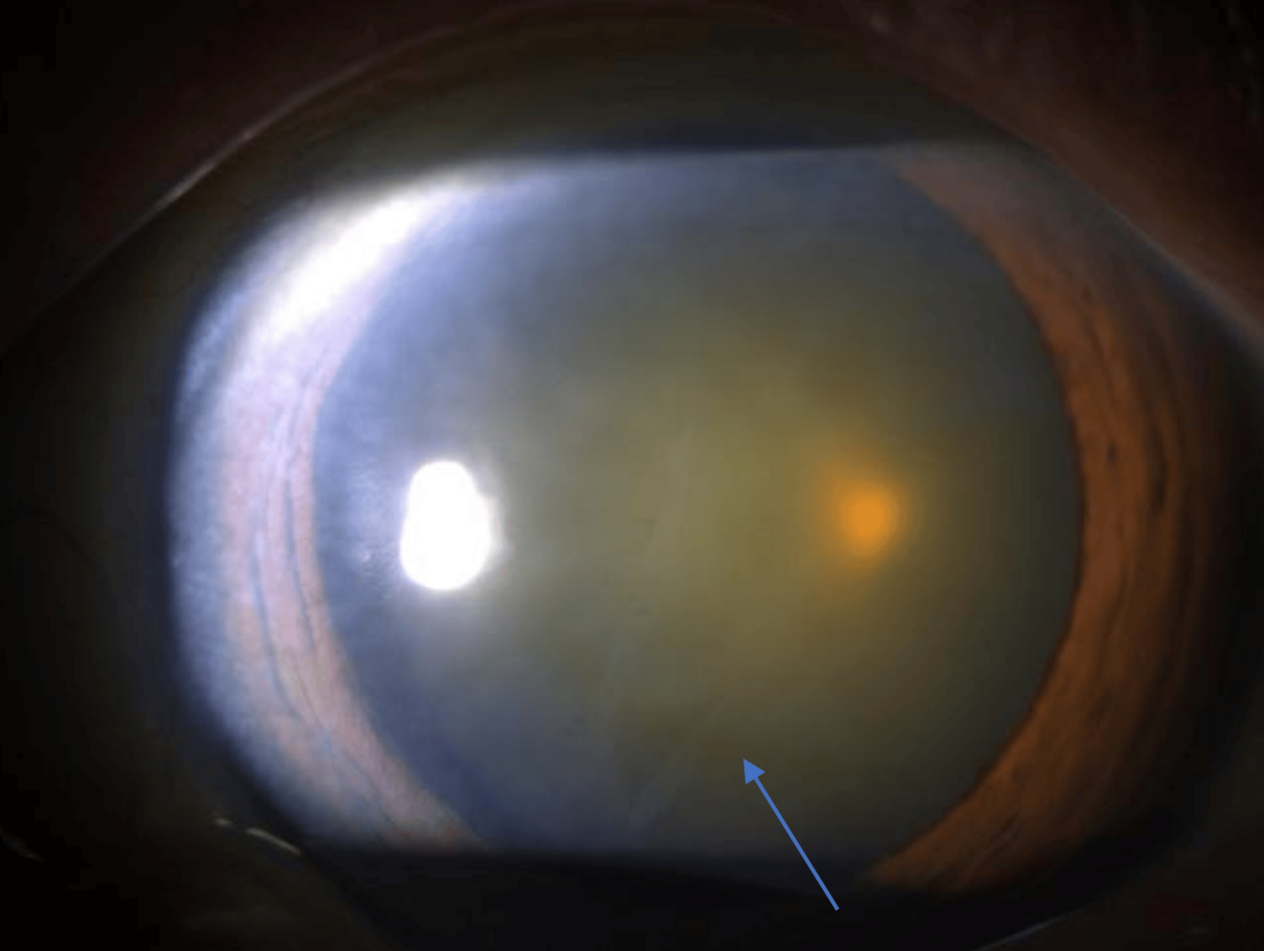
Clinical Photo of the Left Eye Clinical slit lamp photo of the left eye of the patient showing a similar free-floating membrane in the anterior chamber (arrow).

A 75-year-old Chinese woman was referred to our ophthalmology clinic with progressive blurring of vision in both eyes. Her past medical history included hypertension, impaired fasting glucose, and hyperlipidemia. She had no past ophthalmic history. Upon her initial ocular examination, the unaided visual acuities of both eyes were 6/15 by the Snellen chart. The intraocular pressure (IOP) of her right eye was 15 mmHg, and the IOP of her left eye was 16 mmHg. Anterior segment examination showed both eyes with clear corneas and grade 3 nuclear sclerosis cataract. A thin transparent membrane arising from the anterior capsule was also noted floating in the anterior chamber (Figure 1 and Figure 2). There was no iris atrophy nor phacodonesis. No dandruff-like fibrillary materials were found in the anterior chamber on examination. Posterior segment examination was unremarkable with bilateral pink discs and a normal cup-to-disc ratio. Both maculae were normal with flat retinas. Cataract extraction surgery was offered, and the patient was keen to have the left eye cataract extraction first.

**Figure 3:**
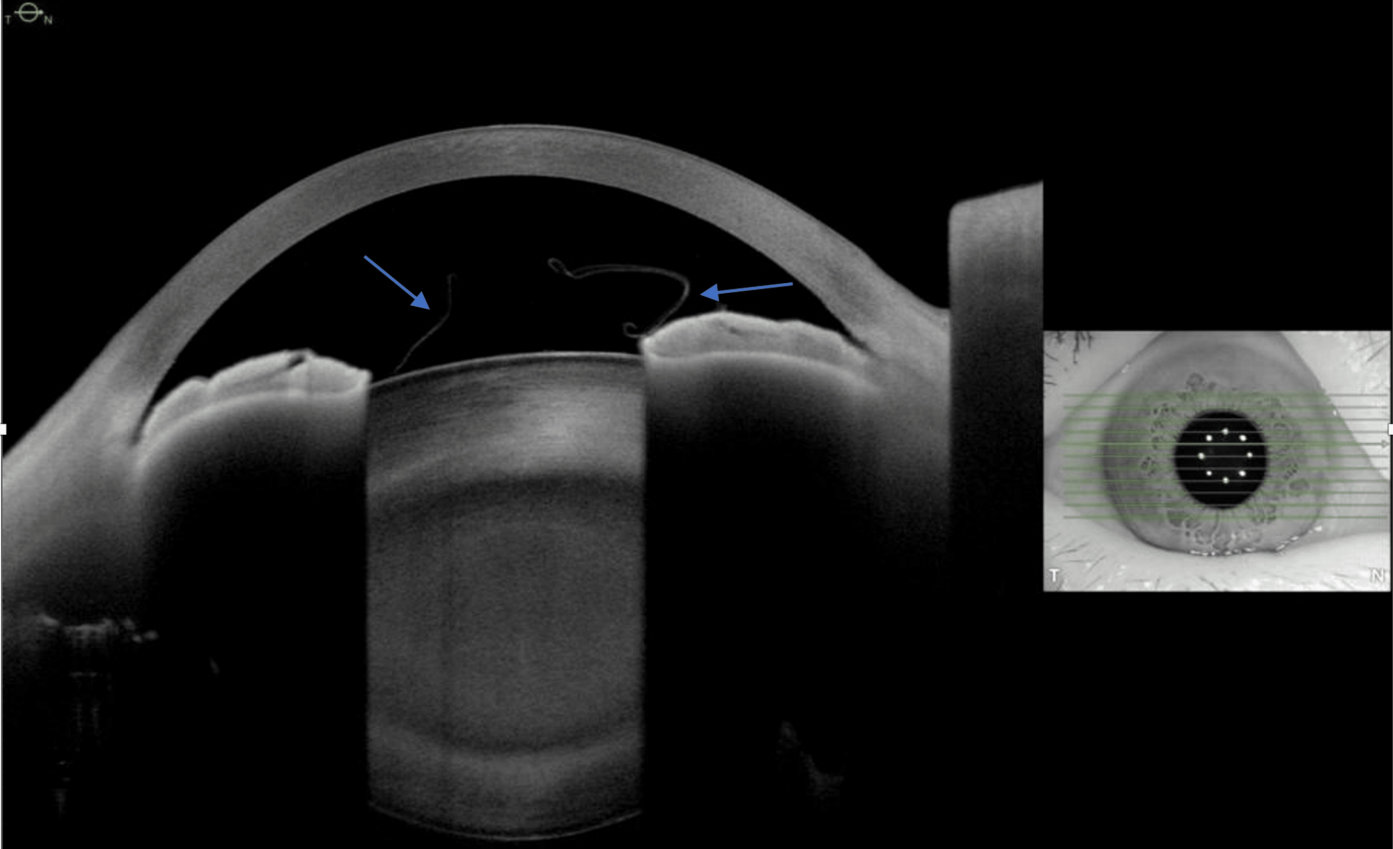
ASCOT Image of the Right Eye ASOCT image of the patient’s right eye showing a refractile, free-floating membrane in the anterior chamber (arrows). ASOCT: anterior segment optical coherence tomography

**Figure 4:**
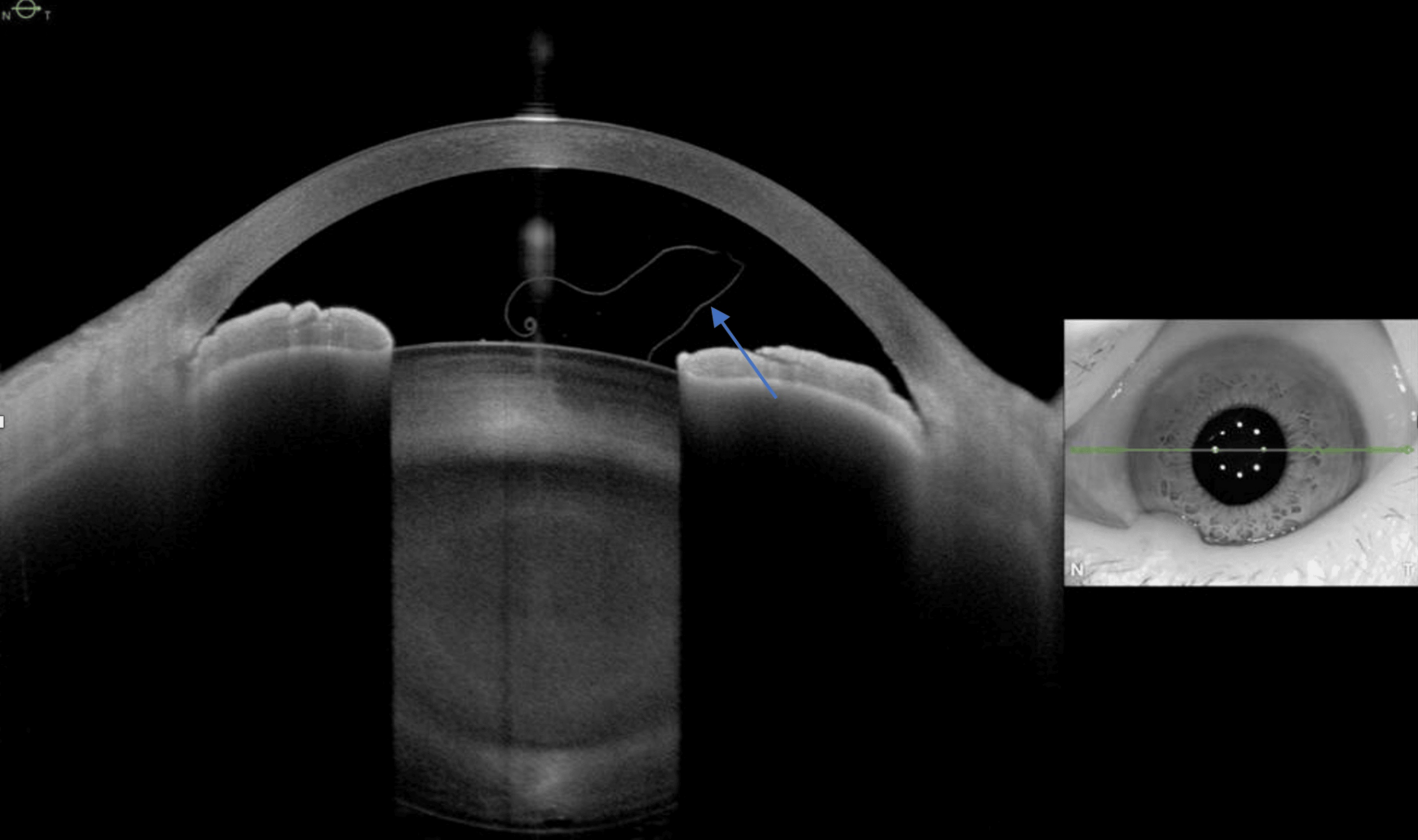
ASCOT Image of the Left Eye ASOCT image of the patient’s left eye showing a refractile, free-floating membrane that curls upon itself in the anterior chamber (arrow). ASOCT: anterior segment optical coherence tomography

**Figure 5:**
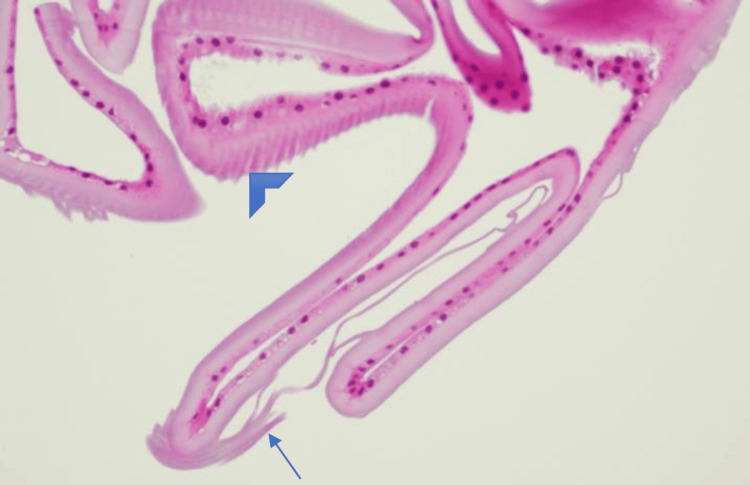
H&E Staining of the Anterior Capsule H&E staining of the anterior capsule showing transitioning from normal thickness (arrowhead) to an abnormally thinned capsule (arrow). H&E: hematoxylin and eosin

**Figure 6:**
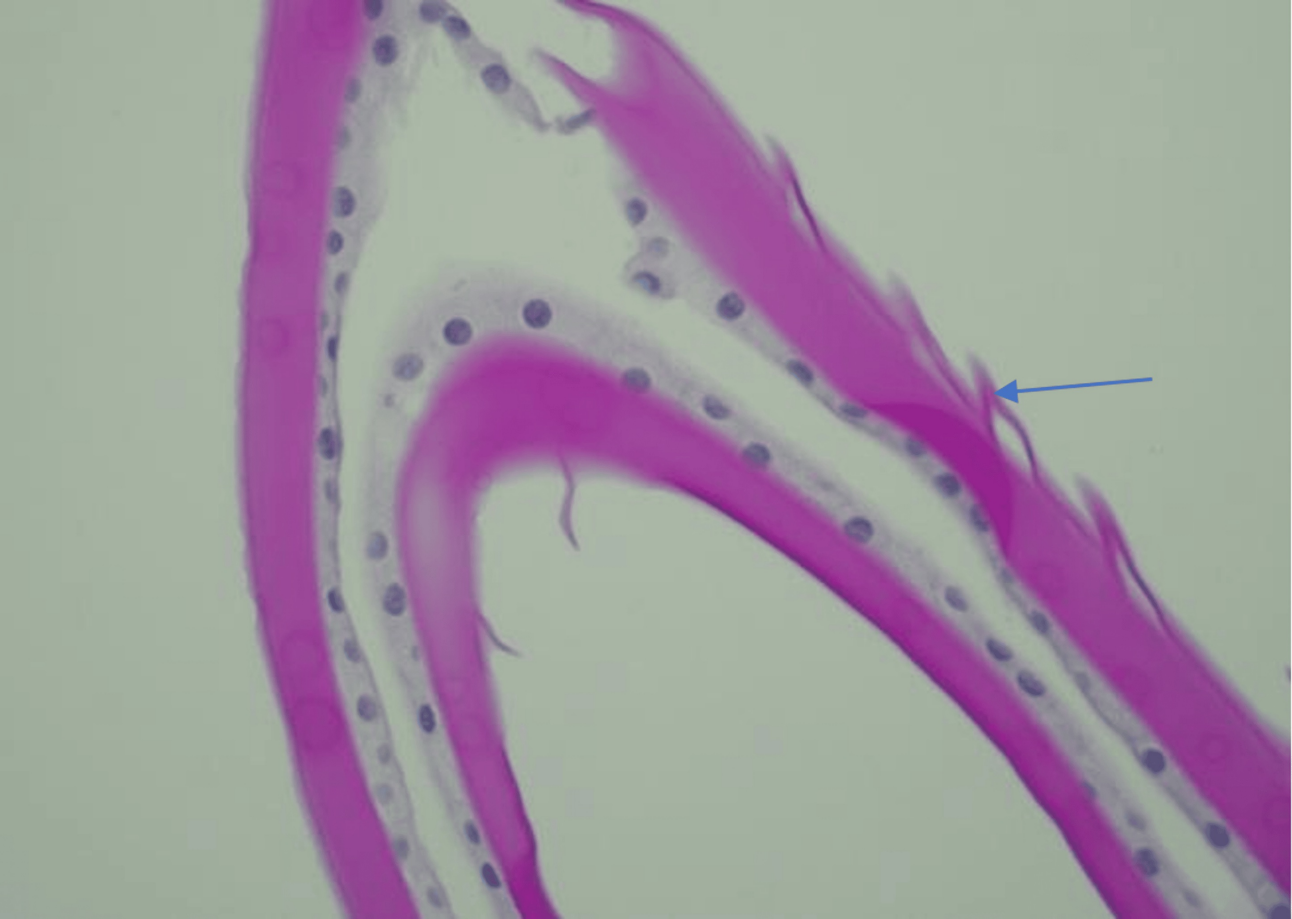
PAS Staining of the Anterior Capsule PAS staining of the anterior capsule showing dehiscence of the capsule. PAS: periodic acid-Schiff

Anterior segment optical coherence tomography (ASOCT) of both eyes were performed and showed a free-floating highly reflective thin membrane in both anterior chambers, respectively (Figure 3 and Figure 4), consistent with delaminated anterior capsules. The patient underwent uneventful left eye phacoemulsification with the use of trypan blue and real-time intraoperative ASOCT, which increased the visibility of the anterior capsule so as to prevent partial capsulorhexis. The anterior capsule with the floating membrane was removed and sent for histological examination. Hematoxylin and eosin (H&E) staining of the anterior capsule showed a transition from a normal-thickness capsule to an abnormally thinned capsule with smaller epithelial cells and reduced cell density (Figure 5). Periodic acid-Schiff (PAS) staining of the anterior capsule demonstrated vesicular degeneration with variable dehiscence of the capsule (Figure 6). The patient was prescribed dexamethasone 0.1% and chloramphenicol 0.5% eyedrops six times per day for one month, and her postoperative recovery was uneventful. The unaided left eye visual acuity improved to 6/9 by the Snellen chart one month after surgery, and her intraocular pressures of both eyes remained normal all along. The patient’s visual acuity remained stable with normal intraocular pressures of both eyes (right eye IOP: 17 mmHg, left eye IOP: 15 mmHg) in her one-year follow-up with no clinical signs of glaucoma.

## Discussion

True exfoliation was first reported by Elschnig in 1922 in glassblowers, historically due to chronic intense heat exposure, which caused lens capsular cell damage due to heat-induced proteolysis of lens capsular protein [1]. Other causes, such as advanced age, intraocular inflammation, ocular trauma, glaucoma, or idiopathic causes, have been attributed to the formation of true exfoliation [2]. The mechanism could be due to direct damage to the capsular cells or indirect initiation of lens capsule proteolysis through enzyme activation [2,3]. It has been proposed that true exfoliation could be more closely associated with age-related degeneration rather than infrared radiation [4]. The pathogenesis is related to vesicle formations in lens capsular cells during aging, in which the vesicles will coalesce to form larger vacuoles, hence leading to anterior lens capsule delamination. It was observed that true exfoliation had a predominance in Asians because of the following postulated mechanism: dark brown irises with the abundance of tropical solar radiation can contribute to increased heat formation, and a shallow anterior chamber with smaller aqueous volumes can result in decreased heat dissipation in the anterior chamber, thus leading to the predisposition of lens capsular damage [5].

True exfoliation can be recognized clinically by the presence of a free-floating, fluttering membrane in the anterior chamber, which represents a split or delaminated anterior capsule. A double ring sign may also be noted intraoperatively, which is the presence of double lines at the capsulorhexis edge [6]. It is believed that a weak point is present between the zonular lamella of the lens and the capsule proper; the tractional force created by capsulorhexis thus leads to further disruption of the delaminated capsule and results in a double ring contour at the capsulorhexis margin [7].

True exfoliation is associated with cataract formation, which could be caused by disturbance in lens metabolism due to the abnormal permeability from a delaminated capsule [5]. The split capsule is prone to capsulorhexis-related complications such as radial extension and radial tear; thus, it is important to recognize features of true exfoliation to facilitate a smooth surgery. Trypan blue-aided capsulorhexis helps in visualizing the capsule status to avoid partial-thickness capsulorhexis [4]. Anterior segment optical coherent tomography (ASOCT) is useful to demonstrate features of true exfoliation, and it is an important preoperative investigation prior to cataract extraction for patients with true exfoliation. A thin and highly refractive membrane resulting from a split anterior lens capsule could be seen on ASOCT. The edge of the membrane could be demonstrated as a free-floating membrane, while the central part of the membrane could be seen adhering to the anterior lens capsule [2]. Therefore, intraoperative real-time ASOCT can help ensure a complete capsulorhexis and avoid partial capsulorhexis. Femtosecond laser-assisted phacoemulsification is promising, in which laser-assisted capsulotomy is noted to be more circular and precise than manual capsulotomy, which, in terms, delivers a better capsule-intraocular lens overlap to reduce posterior capsule opacification and lens decentration [8]. Toledano-Martos et al. reported a patient with age-related true exfoliation undergoing femtosecond laser-assisted phacoemulsification, in which the capsulorhexis was performed with laser uneventfully [9]. A femtosecond laser could indeed be utilized to ensure a more precise and complete capsulorrhexis for patients with true exfoliation undergoing cataract extraction.

## Conclusions

To conclude, true exfoliation is the delamination of the anterior lens capsule. It could be attributed to age-related degeneration and recognized clinically as a free-floating membrane present in the anterior chamber. The fragile delaminated capsule is prone to capsulorhexis-related complications; thus, preoperative and intraoperative anterior segment optical coherence tomography and the utilization of femtosecond laser are all useful to facilitate better surgical planning and execution.
